# The N-terminal disease–associated R5L Tau mutation increases microtubule shrinkage rate due to disruption of microtubule-bound Tau patches

**DOI:** 10.1016/j.jbc.2022.102526

**Published:** 2022-09-24

**Authors:** Alisa Cario, Sanjula P. Wickramasinghe, Elizabeth Rhoades, Christopher L. Berger

**Affiliations:** 1 Department of Molecular Physiology and Biophysics, University of Vermont, Burlington, Vermont, USA; 2 Biochemistry and Molecular Biophysics Graduate Group, Perelman School of Medicine, University of Pennsylvania, Philadelphia, PA, USA; 3 Department of Chemistry, University of Pennsylvania, Philadelphia, PA, USA

**Keywords:** Tau protein, tauopathy, microtubule-associated protein, fluorescence correlation spectroscopy, FRET, TIRF microscopy, microtubule dynamics, ET_eff_, efficiency of energy transfer, FCS, fluorescence correlation spectroscopy, GMPCPP, guanosine-5′-[(α,β)-methyleno]triphosphate, MAP, microtubule-associated protein, PSP, progressive supranuclear palsy, smFRET, single-molecule FRET, TAB, TIRF assay buffer, TIRF, total internal reflection fluorescence

## Abstract

Regulation of the neuronal microtubule cytoskeleton is achieved through the coordination of microtubule-associated proteins (MAPs). MAP-Tau, the most abundant MAP in the axon, functions to modulate motor motility, participate in signaling cascades, as well as directly mediate microtubule dynamics. Tau misregulation is associated with a class of neurodegenerative diseases, known as tauopathies, including progressive supranuclear palsy, Pick's disease, and Alzheimer's disease. Many disease-associated mutations in Tau are found in the C-terminal microtubule-binding domain. These mutations decrease microtubule-binding affinity and are proposed to reduce microtubule stability, leading to disease. N-terminal disease-associated mutations also exist, but the mechanistic details of their downstream effects are not as clear. Here, we investigate the effect of the progressive supranuclear palsy–associated N-terminal R5L mutation on Tau-mediated microtubule dynamics using an *in vitro* reconstituted system. We show that the R5L mutation does not alter Tau interactions with tubulin by fluorescence correlation spectroscopy. Using total internal reflection fluorescence microscopy, we determined that the R5L mutation has no effect on microtubule growth rate, catastrophe frequency, or rescue frequency. Rather, the R5L mutation increases microtubule shrinkage rate. We determine this is due to disruption of Tau patches, larger order Tau complexes known to form on the GDP-microtubule lattice. Altogether, these results provide insight into the role of Tau patches in mediating microtubule dynamics and suggesting a novel mechanism by which mutations in the N-terminal projection domain reduce microtubule stability.

Microtubules are important for a wide array of cellular processes, such as cell division, cell shape, and transport. Proper microtubule function is achieved through orchestration of microtubule-associated proteins (MAPs) in all cell types, but this is especially important in neurons because of the distinct morphology of the axon and necessity for long-range cargo transport. MAP-Tau, the most abundant MAP in the axon, is involved in a variety of microtubule functions. In addition to modulating motor motility (1, 2, 3, 4, 5) and participating in signaling cascades (6, 7), Tau directly regulates microtubule dynamics (8, 9, 10). Tau is known to reduce the critical concentration of tubulin necessary for microtubule polymerization (10, 11). Furthermore, Tau increases microtubule growth rates, decreases shrinkage rates, and reduces catastrophe frequency compared with microtubules in the absence of Tau (10, 11, 12, 13).

The effect of Tau on microtubule dynamics is dependent on Tau association with both the microtubule lattice and free tubulin, mediated by multiple binding sites distributed throughout the C-terminal half of the protein (14). Dependent on alternative splicing of Tau (12, 15, 16, 17), three or four microtubule-binding repeats bind in an extended conformation along a single protofilament (12, 18, 19, 20, 21, 22, 23). The proline-rich region and the pseudo repeat, which flank the microtubule-binding repeats, also contribute to microtubule binding (12, 19, 22, 24). The N-terminal domain of Tau, the projection domain, has the lowest contribution to the overall binding affinity (18, 25). Rather, the projection domain interacts with signaling molecules, other cytoskeletal proteins, and membranes (6, 7, 26, 27, 28).

Tau interaction with the microtubule is complex, as Tau-binding behavior is dependent on Tau concentration and the composition of the microtubule lattice. At low concentrations, on microtubules stabilized with either paclitaxel (Taxol-microtubules) or guanosine-5′-[(α,β)-methyleno]triphosphate (GMPCPP) (GMPCPP-microtubules), Tau binds in a dynamic equilibrium between static and diffusive binding states (29, 30). As the concentration increases, Tau binds nonuniformly and forms larger order complexes comprised of static molecules on Taxol-microtubules but not on GMPCPP-microtubules (30, 31, 32). These complexes *in vitro* have been referred to as Tau patches (3), Tau cohesive islands (33), Tau condensates (31), or Tau envelopes and are important for modulation of motor motility (3, 31). Microtubule-bound Tau complexes have been seen in neurons and other cell types (31, 34, 35). However, the importance of Tau-binding behavior and patch formation on the ability of Tau to regulate microtubule dynamics is unknown.

Tau misregulation and aggregation are linked to a class of neurodegenerative diseases known as ”tauopathies,” which include Alzheimer’s disease, Pick’s disease, and progressive supranuclear palsy (PSP) (36, 37, 38, 39, 40). Many disease-associated mutations in Tau, found within the microtubule-binding region, have been shown to decrease Tau interaction with the microtubule through a decrease in affinity and/or an increased propensity for Tau aggregation (41, 42). Unsurprisingly, these disease-associated mutations also alter the ability of Tau to regulate microtubule dynamics, both in cells and *in vitro* (43, 44, 45).

Tau is known to have a higher affinity for GDP microtubules compared with GTP microtubules (32, 46, 47), and we recently discovered the N-terminal disease–associated mutation in Tau, R5L, associated with PSP, does not reduce affinity for either Taxol-microtubules or GMPCPP-microtubules (32). This is in agreement with past studies that show deletion of the N-terminal domain does not alter Tau affinity for the microtubule (21). Rather, on Taxol-microtubules, the R5L mutation alters Tau microtubule–bound behavior, reducing the occupancy of Tau, and disrupting Tau patches (32). Interestingly, the R5L mutation has been shown to reduce microtubule assembly, but the mechanism remains unclear (45, 48). We hypothesize that the R5L mutation reduces microtubule assembly through disruption of Tau patches on dynamic microtubules. To test this hypothesis, we studied the interactions of WT-Tau and R5L-Tau with both dynamic microtubules and free tubulin, using an *in vitro* reconstituted system. Our results suggest a novel mechanism by which the R5L mutation may lead to disease as well as provide the first evidence for the role of Tau-binding behavior in regulation of microtubule dynamics.

## Results

### The R5L mutation leads to shorter microtubules

**Figure 1:**
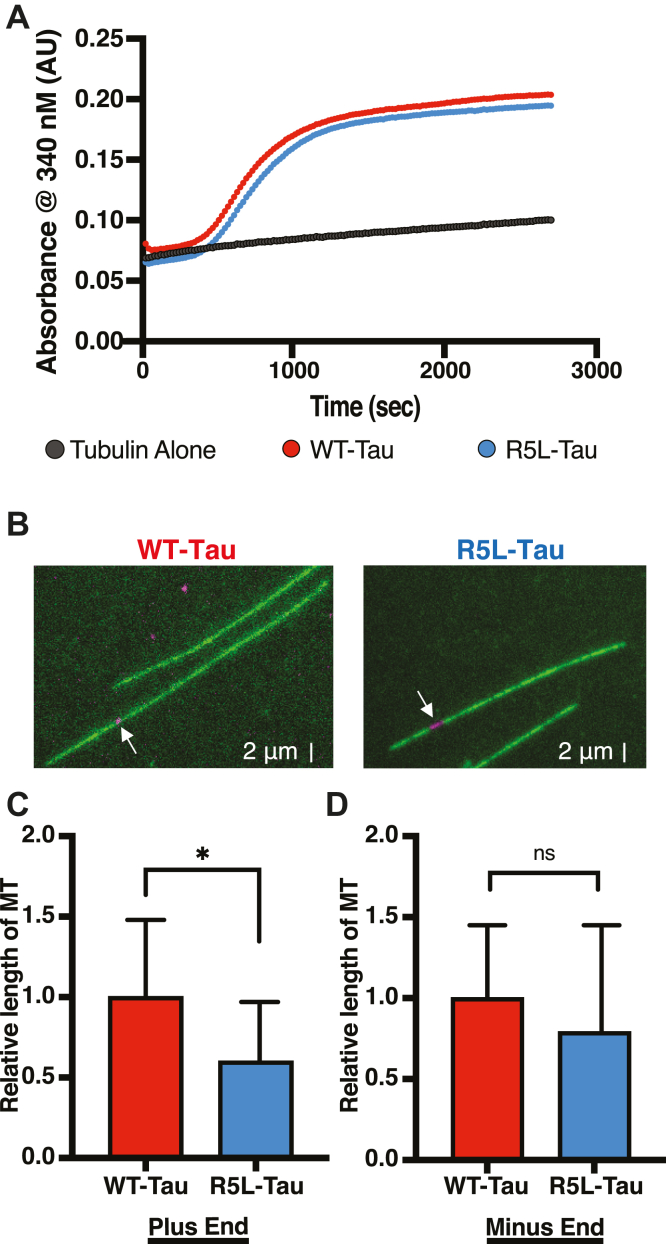
**The R5L mutation leads to shorter plus ends.***A*, microtubule assembly *via* absorbance at 340 nm over time with 20 μM tubulin (tubulin alone, *black*) in the presence of 10 μM WT-Tau (*red*) or R5L-Tau (*blue*). Data are mean (N = 3). *B*, representative images of dynamic microtubules (*green*) grown from GMPCPP seeds (*magenta*) in the presence of WT-Tau (*left*) or R5L-Tau (*right*). *White arrows* indicate GMPCPP seeds. *C*, TIRF assays comparing relative length of microtubule plus ends grown with WT-Tau (*red*, 1.00 ± 0.48, N = 26 microtubules) and R5L-Tau (*blue*, 0.60 ± 0.37, N = 23). Data are mean ± SD. *D*, TIRF assays comparing relative length of microtubule minus ends grown with WT-Tau (*red*, 1.00 ± 0.50, N = 22) and R5L-Tau (*blue*, 0.79 ± 0.66, N = 23 microtubules). Data are mean ± SD. Statistical analysis was performed using Student's *t* test (∗*p* < 0.05). GMPCPP, guanosine-5′-[(α,β)-methyleno]triphosphate; TIRF, total internal reflection fluorescence.

To study the effect of the R5L mutation on Tau-mediated microtubule assembly, the R5L mutation was cloned into the 3RS isoform of Tau; 3RS is lacking both of the N-terminal splice inserts and contains three of four microtubule-binding repeats. Polymerization of microtubules was monitored at 37 °C *via* a bulk light scattering assay using 20 μM tubulin and 10 μM Tau (WT-Tau or R5L-Tau). The R5L mutation does not significantly alter the polymerization lag time or the rate of polymerization (Fig. 1*A*), although there is a slight reduction in the final absorbance.

However, turbidity assays are bulk measurements and cannot differentiate effects of microtubule growth or stabilization. Therefore, the effect of the R5L mutation on Tau-mediated microtubule dynamics was determined using total internal reflection fluorescence (TIRF) microscopy. Microtubules were grown at 37 °C from Alexa 647-labeled GMPCPP seeds with 5 μM Alexa 488 tubulin and 750 nM unlabeled Tau (WT-Tau or R5L-Tau) (Fig. 1*B*), similar to Tau:tubulin ratios used previously, which provide sufficiently long but dynamic microtubules (8, 49). In addition, under these conditions, microtubules will not polymerize without Tau, and therefore, microtubule growth is a Tau-dependent process. The relative length of microtubules from the seed to both plus and minus ends was measured. These relative lengths from the seed are referred to as plus end and minus end, respectively. Dynamic microtubules plus ends grown in the presence of R5L-Tau are approximately 60% of the length of microtubules grown with WT-Tau (WT-Tau = 1.00 ± 0.48; R5L-Tau = 0.60 ± 0.37) (Fig. 1*C*). Yet, the R5L mutation has no effect on minus end length (Fig. 1*D*). Hence, R5L-Tau leads to shorter microtubules relative to WT-Tau because of shorter plus ends.

### The R5L mutation does not alter tau interaction with tubulin

**Figure 2:**
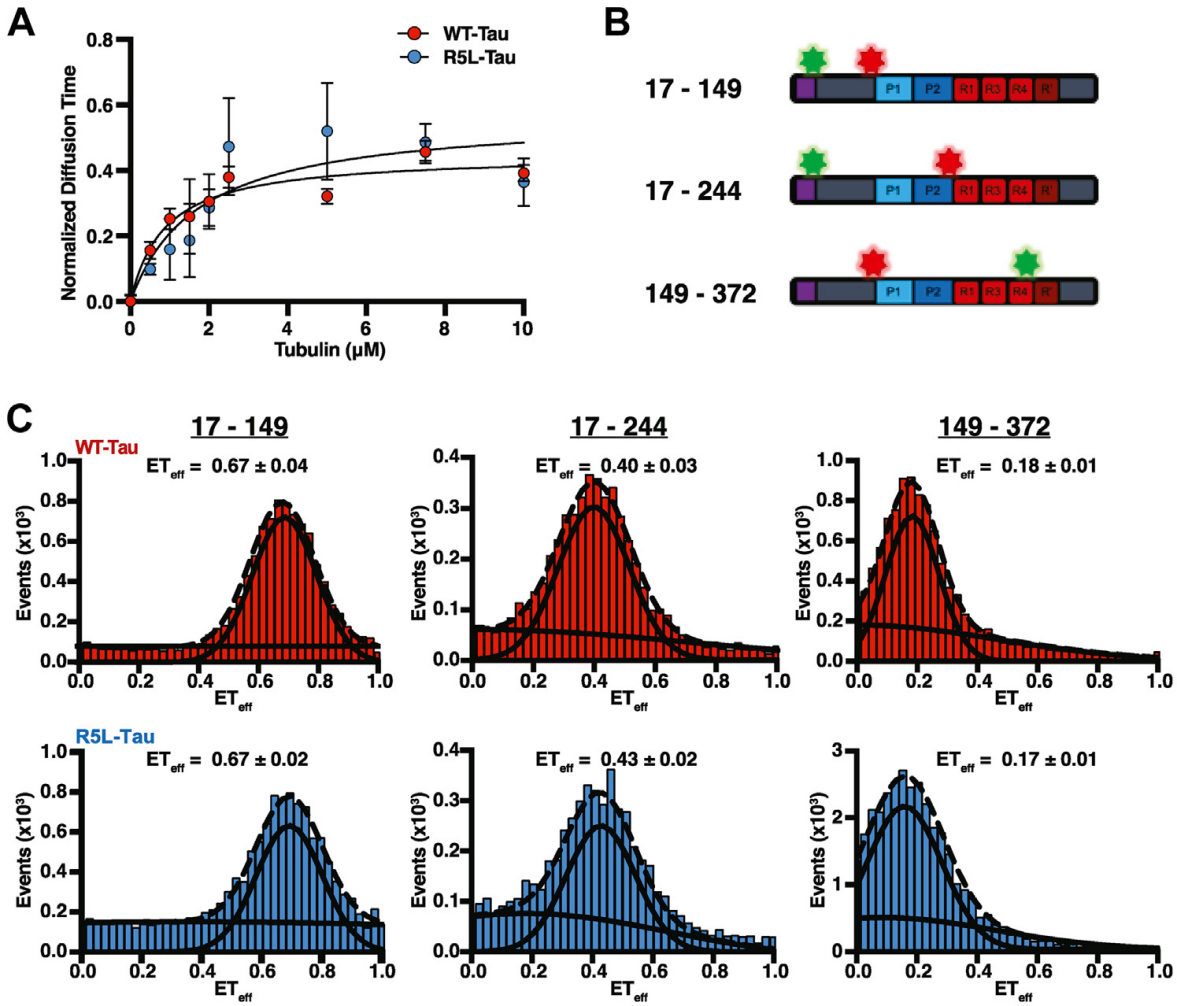
**The R5L mutation does not alter Tau binding to free tubulin.***A*, normalized diffusion times of 20 nM WT-Tau (*red*) or R5L-Tau (*blue*) at varying concentrations of tubulin determined *via* FCS. The data are normalized to the diffusion time in the absence of tubulin and fit to a binding model to determine the relative change in diffusion time at saturating tubulin concentrations. Data are mean ± SD (N = 3). Statistical analysis was performed using Student's *t* test (∗*p* < 0.05). *B*, schematic of Tau-depicting regions probed *via* FRET where the numbers indicate the amino acid number mutated to cysteine for Alexa fluorophore labeling (Alexa 594/488 = *red/green stars*). *C*, FRET efficiency histograms of WT-Tau (*top*, *red*) or R5L-Tau (*bottom*, *blue*) probing various regions within Tau. Residues are numbered based on the longest isoform of Tau. The data were fit with two Gaussians to account for background. Individual (*solid lines*) and sum (*dotted line*) Gaussian fits of representative histograms are shown. There is no difference in FRET efficiency between C17–C149 of WT-Tau (0.65 ± 0.02) and R5L-Tau (0.67 ± 0.02). There is no difference in FRET efficiency between C17–C244 of WT-Tau (0.36 ± 0.01) and R5L-Tau (0.41 ± 0.02). There is no difference in FRET efficiency between C149–C372 of WT-Tau (0.17 ± 0.003) and R5L-Tau (0.17 ± 0.001). Data are mean ± SEM (N = 3). Statistical analysis was performed using Student's *t* test (∗*p* < 0.05). FCS, fluorescence correlation spectroscopy.

Shorter microtubules could result from any combination of altered interactions with tubulin, the GTP microtubule lattice, and/or the GDP microtubule lattice. To test if the R5L mutation alters Tau binding to tubulin, fluorescence correlation spectroscopy (FCS) was used to study Tau:tubulin interactions under nonpolymerizing conditions. Diffusion times of 20 nM Alexa 488-labeled Tau (WT-Tau or R5L-Tau) were measured with increasing concentrations of tubulin (0.5–10 μM). The normalized diffusion times of both WT-Tau and R5L-Tau increase by 50% in the presence of saturating concentrations of Tau (Fig. 2*A*), indicating no difference in the Tau:tubulin binding ratio upon introduction of the R5L mutation.

However, in the absence of tubulin, R5L-Tau has a larger diffusion time compared with WT-Tau (WT-Tau = 0.59 ± 0.01 ms; R5L-Tau = 0.67 ± 0.01 ms) (Fig. S1). The change in diffusion time may be due to structural changes within Tau upon introduction of the mutation. Tau is an intrinsically disordered protein but can adopt an ensemble of transient global conformations in solution (25, 50). Therefore, single-molecule FRET (smFRET) was used to investigate the possible effects of the R5L mutation on the conformation of Tau. Differences in FRET efficiencies (efficiency of energy transfer [ET_eff_]), calculated from the ratio of intensity of the acceptor over the sum of the intensities of the donor and acceptor fluorophores, indicate relative changes in distance between regions within Tau, which can provide insight into the global conformation of the protein. Amino acids were mutated to cysteine residues to conjugate fluorophores *via* maleimide chemistry, and the numbering used is relative to the longest Tau isoform. The R5L mutation has no effect on the FRET efficiency within the N-terminal projection domain (C17–C149) of Tau (WT-Tau = 0.67 ± 0.04; R5L-Tau = 0.67 ± 0.02). Likewise, the R5L mutation does not alter the observed distance between the projection domain and the central proline-rich region (C17–C244) (WT-Tau = 0.40 ± 0.03; R5L-Tau = 0.43 ± 0.02) or between the proline-rich region and the microtubule-binding repeats (C149–C372) (WT-Tau = 0.18 ± 0.01; R5L-Tau = 0.17 ± 0.01) (Fig. 2*C*). The ET_eff_ values are consistent with our previous work of Tau conformations in solution using FRET (24, 51). However, it is possible there are changes below the FRET detection limit for this probe pair at the positions chosen. Therefore, local structural changes near the R5L mutation, which have been previously observed by NMR (32), could account for the changes in diffusion times.

### The R5L mutation increases the microtubule shrinkage rate

**Figure 3:**
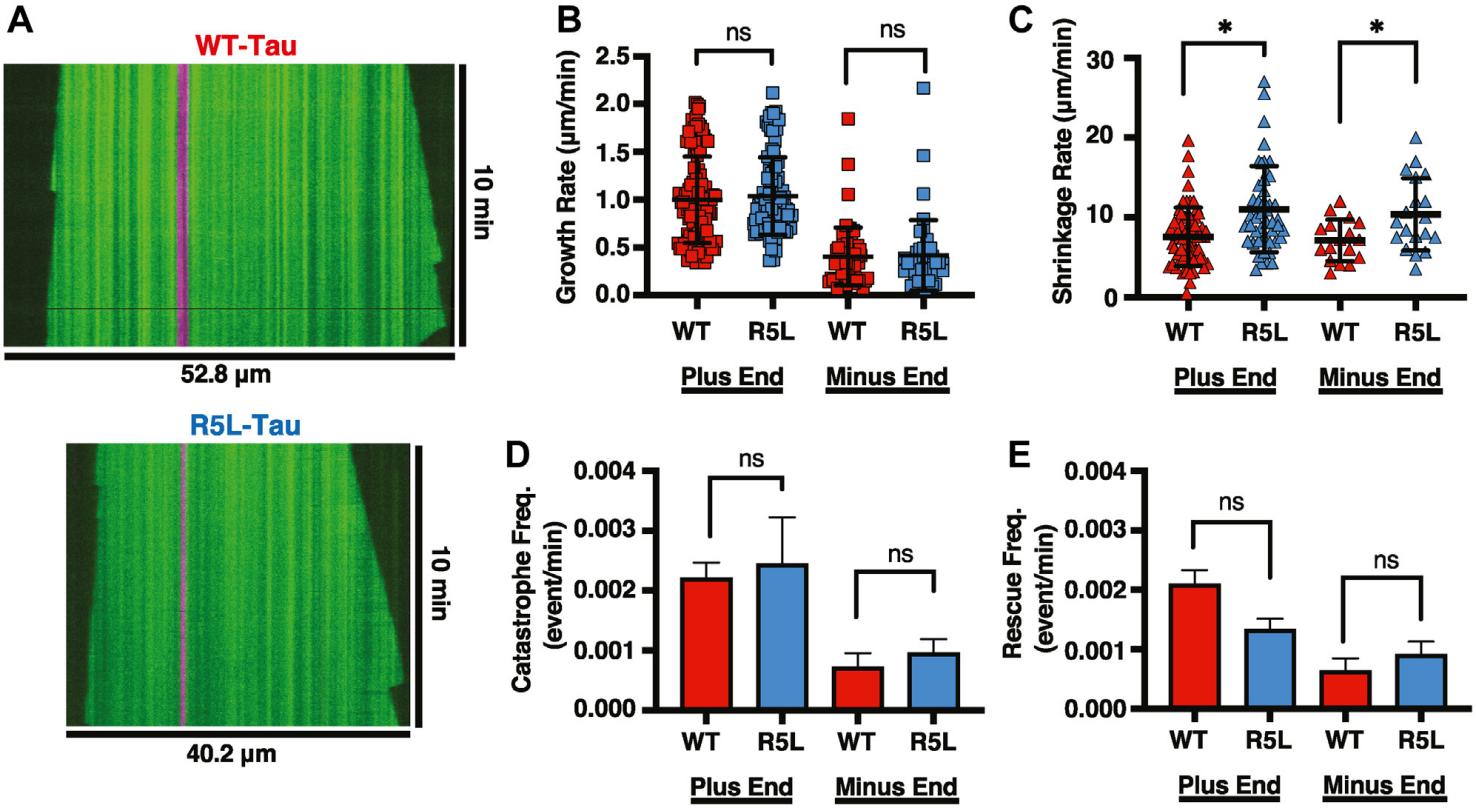
**The R5L mutation increases microtubule shrinkage rate.***A*, representative kymographs of dynamic microtubules (*green*) grown from GMPCPP seeds (*magenta*) in the presence of 750 nM WT-Tau (*top*) or R5L-Tau (*bottom*). *B*, microtubule growth rate at plus end comparing WT-Tau (*red*, 1.00 ± 0.4 μm/min, N = 106 events) and R5L-Tau (*blue*, 1.04 ± 0.4 μm/min, N = 120 events) and minus end comparing WT-Tau (*red*, 0.40 ± 0.30 μm/min, N = 63 events) and R5L-Tau (*blue*, 0.41 ± 0.37 μm/min, N = 44 events). Data are mean ± SD. Statistical analysis was performed using Welch’s test (∗*p* < 0.05). *C*, microtubule shrinkage rate at plus end comparing WT-Tau (*red*, 7.55 ± 3.65 μm/min, N = 65 events) and R5L-Tau (*blue*, 11.02 ± 5.39 μm/min, N = 46 events) and minus end comparing WT-Tau (*red*, 7.12 ± 2.60 μm/min, N = 19 events) and R5L-Tau (*blue*, 10.37 ± 4.53 μm/min, N = 17 events). Data are mean ± SD. Statistical analysis was performed using Welch’s test (∗*p* < 0.05). *D*, microtubule catastrophe frequency at plus end comparing WT-Tau (*red*, 0.002 ± 0.001 events/min, N = 56 microtubules) and R5L-Tau (*blue*, 0.002 ± 0.001 events/min, N = 60 microtubules) and minus end comparing WT-Tau (*red*, 0.0007 ± 0.0002 events/min, N = 33 microtubules) and R5L-Tau (*blue*, Tau 0.0009 ± 0.0092, N = 37 microtubules). Data are mean ± SEM. Statistical analysis was performed using Welch’s test (∗*p* < 0.05). *E*, microtubule rescue frequency at plus end comparing WT-Tau (*red*, 0.002 ± 0.001 events/min, N = 54 microtubules) and R5L-Tau (*blue*, 0.001 ± 0.001 events/min, N = 48 microtubules) and minus end comparing WT-Tau (*red*, 0.0007 ± 0.0002, N = 33 microtubules) and R5L-Tau (*blue*, 0.0009 ± 0.0092, N = 42 microtubules). Data are mean ± SEM. Statistical analysis was performed using Welch’s test (∗*p* < 0.05). GMPCPP, guanosine-5′-[(α,β)-methyleno]triphosphate.

To further understand how the R5L mutation leads to shorter microtubules and altered interaction with the microtubule lattice, microtubule growth rate, shrinkage rate, catastrophe, and rescue frequency were measured in the presence of WT-Tau or R5L-Tau using TIRF microscopy (Movie S1 and S2). Relative to WT-Tau, R5L-Tau has no effect on microtubule growth rate at either the plus end (WT-Tau = 1.00 ± 0.4 μm/min; R5L-Tau = 1.04 ± 0.4 μm/min) or the minus end (WT-Tau = 0.40 ± 0.30 μm/min; R5L-Tau = 0.41 ± 0.37 μm/min) (Fig. 3*B*). Similarly, there is no difference in the amount of time microtubules are growing or shrinking (Fig. S2). In addition, there is no difference in the catastrophe frequency at the plus end (WT-Tau = 0.002 ± 0.001 events/min; R5L-Tau = 0.002 ± 0.001 events/min) or the minus end (WT-Tau = 0.0007 ± 0.0002 events/min; R5L-Tau 0.0009 ± 0.0092 events/min) (Fig. 3*D*). Similarly, there is no difference in the rescue frequency at the plus end (WT-Tau = 0.002 ± 0.001 events/min; R5L-Tau = 0.001 ± 0.001 events/min) or the minus end (WT-Tau = 0.0007 ± 0.0002 events/min; R5L-Tau = 0.0009 ± 0.0092 events/min) (Fig. 3*E*). However, compared with WT-Tau, R5L-Tau increases the Tau-mediated microtubule shrinkage rate at the plus end (WT-Tau = 7.55 ± 3.65 μm/min; R5L-Tau = 11.02 ± 5.39 μm/min) and the minus end (WT-Tau = 7.12 ± 2.60 μm/min; R5L-Tau = 10.37 ± 4.53 μm/min) (Fig. 3*C*).

### The reduction in occupancy upon introduction of the R5L mutation does not alter microtubule shrinkage rate

**Figure 4:**
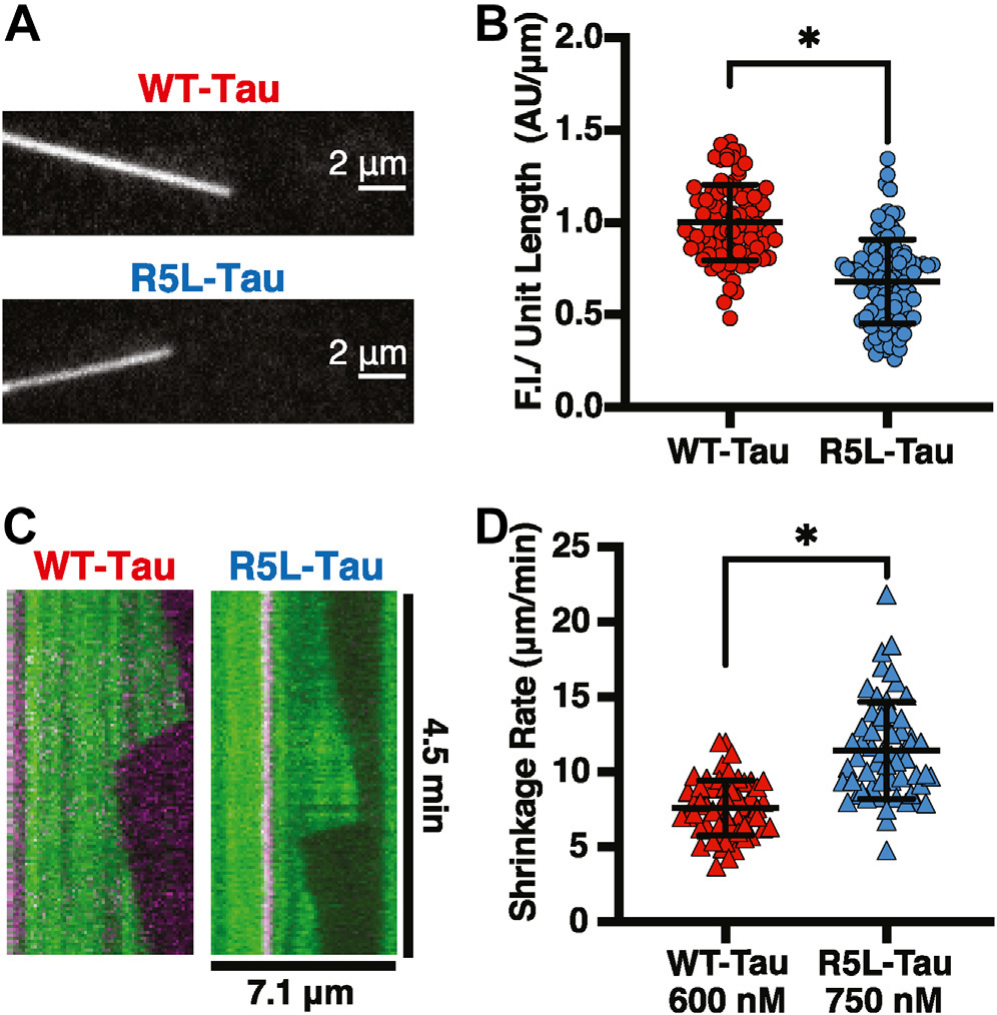
**Increased microtubule shrinkage rate is not a result of reduction in Tau occupancy on the microtubule lattice.***A*, representative images of 750 nM labeled WT-Tau (*top*) or R5L-Tau (*bottom*) on dynamic microtubules. *B*, TIRF assays comparing relative normalized fluorescence intensity of WT-Tau (*red*, 1.00 ± 0.20, N = 95 microtubules) and R5L-Tau (*blue*, 0.68 ± 0.23, N = 101 microtubules). Data are mean ± SD. Statistical analysis was performed using Student's *t* test (∗*p* < 0.05). *C*, representative kymographs of dynamic microtubules (*green*) grown from GMPCPP seeds (*magenta*) at the same amount of Tau bound, 600 nM WT-Tau (*left*) and 750 nM R5L-Tau (*right*). *D*, microtubule shrinkage rate at same amount of Tau bound, 600 nM WT-Tau (*red*, 7.62 ± 1.84 μm/min, N = 59 events) and 750 nM R5L-Tau (*blue*, 11.46 ± 3.24 μm/min, N = 58 events). Data are mean ± SD. Statistical analysis was performed using Welch’s test (∗*p* < 0.05). GMPCPP, guanosine-5′-[(α,β)-methyleno]triphosphate; TIRF, total internal reflection fluorescence.

The R5L mutation increases microtubule shrinkage rate indicating the R5L mutation alters Tau’s interaction with the GDP lattice, as depolymerizing microtubules are comprised of GDP tubulin (52, 53). Recently, we studied the effect of R5L mutation on the ability of Tau to interact with stabilized microtubules. On paclitaxel stabilized microtubules (Taxol-microtubules), a mimic of the GDP lattice, the R5L mutation reduces the occupancy of Tau, or the amount of Tau bound at saturating conditions without altering affinity, shown using both single molecule and bulk fluorescence assays over a range of Tau concentrations (32). We hypothesize that the reduction in occupancy of R5L-Tau, relative to WT-Tau, leads to an increase in microtubule shrinkage rate. Therefore, the amount of Tau (WT-Tau or R5L-Tau) bound to dynamic microtubules was determined by measuring the normalized fluorescence intensity of 750 nM Alexa 647-labeled Tau (WT-Tau or R5L-Tau) on Alexa 488-labeled dynamic microtubules. Similar to previous work on Taxol-microtubules, under these conditions, R5L-Tau has approximately two-third the amount of Tau bound compared with WT-Tau (Fig. 4*B*) (32). To determine if the reduction in occupancy of R5L-Tau is leading to an increase in microtubule shrinkage rate, the concentration of WT-Tau was reduced to equal the amount of R5L-Tau bound, and microtubule shrinkage rate was measured. At the same amount of Tau bound, (600 nM WT-Tau and 750 nM R5L-Tau), determined by normalized fluorescence intensity, R5L-Tau has a faster shrinkage rate compared with WT-Tau (WT-Tau = 7.62 ± 1.84 μm/min; R5L-Tau = 11.46 ± 3.24 μm/min) (Fig. 4*D*). Therefore, the reduction in occupancy is not the cause of the increase in Tau-mediated microtubule shrinkage rate upon introduction of the mutation.

### Tau patches form on dynamic microtubules

**Figure 5:**
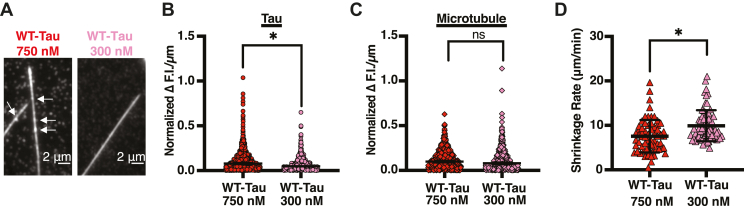
**Tau patches correlate with a decrease in microtubule shrinkage rate.***A*, representative images of 750 nM WT-Tau (*left*) and 300 nM WT-Tau (*left*) indicating Tau patches. *White arrows* indicate Tau patches. *B*, heterogeneity of Tau fluorescence intensity along dynamic microtubules comparing 750 nM WT-Tau (*red*, 0.08 ± 0.006 Δ F.I./μm, N = 1228 measurements) and 300 nM WT-Tau (*pink*, 0.048 ± 0.009 Δ F.I./μm, N = 482 measurements). Data are median ± 95% confidence interval (CI). Statistical analysis was performed using Mann–Whitney test (∗*p* < 0.001). *C*, heterogeneity of dynamic microtubule fluorescence intensity comparing microtubules grown with 750 nM WT-Tau (*red*, 0.10 ± 0.09 Δ F.I./μm, N = 775 measurements) or 300 nM WT-Tau (*pink*, 0.08 ± 0.01 Δ F.I./μm, N = 482 measurements). Data are median ± 95% CI. Statistical analysis was performed using Mann–Whitney test (∗*p* < 0.001). *D*, microtubule shrinkage rate comparing 750 nM WT-Tau (*red*, 7.55 ± 3.66, N = 65 events) and 300 nM WT-Tau-Tau (*pink*, 9.91 ± 3.51, N = 71 events). Data are mean ± SD. Statistical analysis was performed using Welch’s test (∗*p* < 0.05).

However, the R5L mutation also disrupts Tau-binding behavior on Taxol-microtubules. Previous work has shown at low concentrations, Tau binds to Taxol-microtubules in an equilibrium between static and diffusive binding states (29, 30). However, as the concentration of Tau increases, so does the formation of static Tau patches (3, 30, 31, 33). It is not known whether Tau patches form on dynamic microtubules or if they have a role in mediating microtubule dynamics. Therefore, to determine if Tau patches form on dynamic microtubules, microtubules were polymerized with either 300 nM or 750 nM WT-Tau (100 nM Alexa 647-labeled and either 200 nM or 650 nM unlabeled WT-Tau) and 5 μM Alexa 488-labeled tubulin. Tau patch formation was determined by measuring the heterogeneity of Tau fluorescence along the length of the microtubule. The use of Tau fluorescence heterogeneity as a measurement for patch formation was confirmed using stabilized microtubules, as Tau patches form on Taxol-microtubules but not GMPCPP-microtubules (30, 31). As expected, Tau binds more heterogeneously to Taxol-microtubules compared with GMPCPP-microtubules (Fig. S3). On dynamic microtubules, 750 nM WT-Tau binds more heterogeneously compared with 300 nM WT-Tau, suggesting WT-Tau forms concentration-dependent Tau patches on dynamic microtubules (Fig. 5*B*). To determine a correlation between Tau patches and shrinkage rate, microtubule shrinkage rate was measured from microtubules polymerized in the presence of 300 nM or 750 nM WT-Tau and 5 μM Alexa 488-labeled tubulin. Compared with 750 nM WT-Tau, 300 nM WT-Tau has a faster microtubule shrinkage rate (750 nM WT-Tau = 7.55 ± 3.65 μm/min; 300 nM WT-Tau = 9.91 ± 3.51 μm/min) (Fig. 5*D*), consistent with Tau patches playing a role in decreasing microtubule shrinkage rate. Furthermore, the microtubule shrinkage rate can be seen to fluctuate at the plus end of dynamic microtubules in the presence of 750 nM WT-Tau, slowing in regions of high Tau concentration, indicative of Tau patches (Fig. S4).

### The R5L mutation disrupts formation of static tau patches on dynamic microtubules

**Figure 6:**
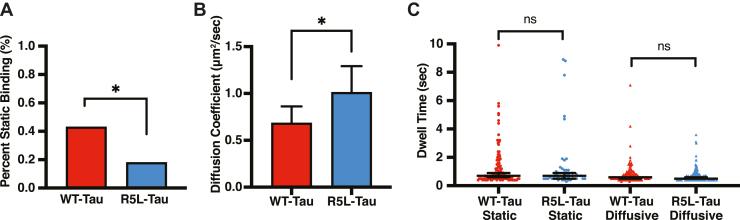
**The R5L mutation decreases the proportion of static Tau-binding events.***A*, percent static binding of 750 nM WT-Tau (*red*) and R5L-Tau (*blue*) on dynamic microtubules. WT-Tau binds statically 43% (N = 267 events) and R5L-Tau binds 18% (N = 253 events). Statistical analysis was performed using Fisher’s exact test (∗*p* < 0.01). *B*, diffusion coefficient of WT-Tau (*red*, 0.68 ± 0.18 μm^2^/s, N = 152 events) or R5L-Tau (*blue*, 1.01 ± 0.28 μm^2^/s, N = 208 events). Data are median ± 95% confidence interval (CI). Statistical analysis was performed using Mann–Whitney test (∗*p* < 0.01). *C*, dwell times of WT-Tau (*red*) and R5L-Tau (*blue*). WT-Tau static events have a dwell time of 0.7 ± 0.2 s (N = 115 events). R5L-Tau static events have a dwell time of 0.7 ± 0.2 s (N = 45 events). WT-Tau diffusive events have a dwell time of 0.6 ± 0.1 s (N = 15 events). R5L-Tau diffusive events have a dwell time of 0.5 ± 0.1 s (N = 208 events). Data are median ± 95% confidence interval (CI). Statistical analysis was performed using Mann–Whitney test (∗*p* < 0.01).

Recently, we showed the R5L mutation disrupts the ability of Tau to form patches on Taxol-microtubules (32). Therefore, we hypothesize that the R5L mutation disrupts Tau patches on dynamic microtubules, and this shift in binding behavior leads to an increase in microtubule shrinkage rate. To test whether the R5L mutation disrupts static Tau patches, Tau-binding behavior was measured using TIRF microscopy. Dynamic microtubules were grown with 5 μM Alexa 488-labeled tubulin and 750 nM Tau (WT-Tau or R5L-Tau) spiked with 300 pM Alexa 647-labeled Tau to visualize individual Tau molecules (Movie S3 and S4). The static:diffusive equilibrium, diffusion coefficient, and dwell times of both static- and diffusive-bound molecules were measured on the dynamic microtubules. The R5L mutation shifts Tau binding toward a diffusive state, similar to what was seen previously on Taxol-microtubules. R5L-Tau binds with 18% of molecules in static state compared with WT-Tau with 43% of statically bound molecules (Fig. 6*A*). Representative kymographs are shown (Fig. S5). Compared with WT-Tau, R5L-Tau has a larger diffusion coefficient (WT-Tau = 0.68 ± 0.18 μm^2^/s; R5L-Tau = 1.01 ± 0.29 μm^2^/s) (Fig. 6*B*). However, there is little overall difference in the dwell times of either static-bound (WT-Tau = 0.7 ± 0.2 s; R5L-Tau = 0.7 ± 0.2 s) or diffusive-bound (WT-Tau = 0.6 ± 0.1 s; R5L-Tau = 0.5 ± 0.1 s) molecules (Figs. 6*C* and S6).

**Figure 7:**
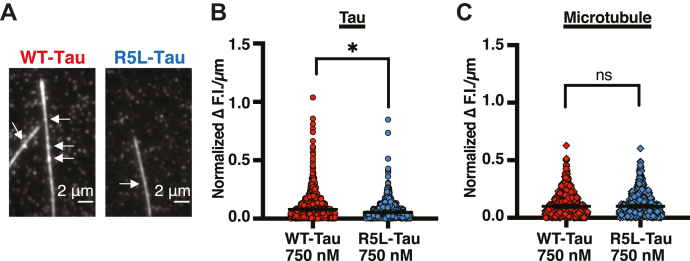
**The R5L mutation disrupts Tau patches.***A*, representative images of 750 nM WT-Tau (*left*, as shown in Fig. 5) and 750 nM R5L-Tau (*right*) indicating Tau patches. *White arrows* indicate Tau patches. *B*, heterogeneity of Tau fluorescence intensity along dynamic microtubules comparing 750 nM WT-Tau (*red*, 0.08 ± 0.006 Δ F.I./μm, N = 1228 measurements) and 750 nM R5L-Tau-Tau (*blue*, 0.053 ± 0.005 Δ F.I./μm, N = 936 measurements). Data are median ± 95% confidence interval (CI). Statistical analysis was performed using Mann–Whitney test (∗*p* < 0.001). *C*, heterogeneity of dynamic microtubule fluorescence intensity comparing microtubules grown with WT-Tau (*red*, 0.10 ± 0.09 Δ F.I./μm, N = 775 measurements) or R5L-Tau (*blue*, 0.10 ± 0.01 Δ F.I./μm, N = 691 measurements). Data are median ± 95% confidence interval (CI). Statistical analysis was performed using Mann–Whitney test (∗*p* < 0.001).

To study R5L-Tau patches directly, dynamic microtubules were polymerized with 5 μM Alexa 488-labeled tubulin, 100 nM Alexa 647-labeled R5L-Tau, and 650 nM unlabeled R5L-Tau. Consistent with our previous work on Taxol-microtubules, R5L-Tau binds more uniformly compared with WT-Tau on dynamic microtubules, suggesting R5L-Tau binds as predominantly single molecules (Fig. 7*B*). It was confirmed that this is not because of fluctuations of the microtubule in the TIRF field as the fluorescence heterogeneity of microtubules does not differ between WT-Tau and R5L-Tau (Fig. 7*C*). Taken together with the change in the behavior of individual molecules, these data support our hypothesis that disruption of Tau patches by the R5L mutation leads to an increase in microtubule shrinkage rate.

## Discussion

In this study, we examine the effect of the PSP-associated R5L Tau mutation on Tau-mediated microtubule dynamics. Previous work has shown that the R5L mutation reduces overall microtubule assembly (54), despite the mutation being located in the N-terminal projection domain, well outside the microtubule-binding repeats. Unsurprisingly, this is not because of a reduction in Tau affinity for microtubules (32). Here, we use single-molecule approaches to build upon the previous bulk turbidity assays (54) and show that the main effect of the R5L mutation is to increase Tau-mediated microtubule shrinkage rate at both the plus and minus ends. Although our assays only indicate shorter microtubule plus ends with the R5L mutation, minus ends are inherently less dynamic than plus ends (52, 55, 56, 57) and therefore less sensitive to changes in microtubule shrinkage rate.

Tau is an intrinsically disordered protein but adopts conformations where the N and C termini interact in solution (25, 50, 58). The FCS experiments indicate a difference in diffusion time upon introduction of the mutation in the absence of tubulin, suggesting a potential structural change. This difference was not observed in the ET_eff_ values between WT-Tau and R5L-Tau across various regions probed for structural changes *via* FRET. This is in agreement with our recent study using NMR, showing that the R5L mutation alters the local structure of Tau, 10 amino acids from the site of the mutation (32). Therefore, the change in diffusion time between WT-Tau and R5L-Tau is potentially because of local structural changes that are undetectable *via* FRET, at least for the positions chosen. Taken together, this further supports that the R5L mutation does not alter global Tau structure.

This work indicates that the R5L mutation does not alter microtubule growth rate, consistent with the lack of an effect of the R5L mutation on Tau binding with tubulin and the GTP microtubule. For microtubules to polymerize or grow, new tubulin dimers must be added to the growing end of the microtubule, known as the GTP cap (52, 59). Tau is known to increase the growth rate of microtubules, which is thought to occur by increasing the local concentration of tubulin through interactions with multiple microtubule-binding repeats of Tau (9). Recently, we showed that the R5L mutation does not alter the affinity or binding behavior of Tau on GTP lattice mimic, GMPCPP-microtubules (32), consistent with the inability of the R5L mutation to affect growth rate. Furthermore, in this study, it was determined that the R5L mutation does not alter Tau-binding stoichiometry to soluble tubulin. Taken together, the data showing the R5L mutation does not alter Tau-mediated microtubule growth rate suggest that there is no effect of the mutation on interactions with soluble tubulin or the GTP microtubule lattice.

Similarly, the R5L mutation does not have an effect on Tau-mediated catastrophe frequency. It is known for a catastrophe to occur, the microtubule loses the GTP cap, and the newly exposed GDP tubulin weakens longitudinal and lateral bonds between protofilaments, leading to depolymerization (60). A large factor in promoting catastrophe frequency is the age of the microtubules (61, 62), which does not differ between WT-Tau and R5L-Tau in these experiments. Although the mechanism remains unknown, Tau is known to reduce the catastrophe frequency of microtubules (9). One potential mechanism is that Tau increases the size of the GTP cap, as Tau also increases the growth rate of microtubules (9). However, a reduction in catastrophe frequency does not always correlate with a larger GTP cap, which has been shown for XMAP215 (63). Other potential explanations for Tau reducing catastrophe frequency are altering the end structure of the microtubule or slowing down the rate of GTP hydrolysis. However, in all these models, Tau reduces catastrophe frequency through interactions with the GTP microtubule lattice. Therefore, it is not surprising that the R5L mutation does not change the catastrophe frequency as the R5L mutation does not alter Tau interaction with the GTP lattice.

Models of Tau-mediated microtubule dynamics predict an effect on microtubule rescue (8, 46, 49). Although still poorly understood, rescue events, transitions from microtubule depolymerization to polymerization, are thought to occur when depolymerization is disrupted. It has been proposed that this may be due to GTP islands within the GDP lattice (64, 65), which would predict no effect of the R5L mutation as R5L-Tau does not alter Tau binding to GTP-microtubules. Indeed, introduction of the R5L mutation does not lead to a statistically significant change in rescue frequency. However, it should be noted that there is a trend toward fewer rescues with R5L-Tau relative to WT-Tau (Fig. 3*E*). It is known that the presence of Tau reduces the dynamicity of microtubules or the number of catastrophes and subsequent rescues (8). Furthermore, rescues are highly efficient in the presence of Tau and typically occur after each catastrophe. Therefore, an effect of the R5L mutation on Tau-mediated microtubule rescue cannot be ruled out. In fact, a reduction in rescue frequency upon introduction with the R5L mutation is not inconsistent with our understanding of how the R5L mutation disrupts Tau binding on the GDP lattice, which is discussed later.

Recently, we discovered that the R5L mutation had two effects on Tau binding to GDP lattice mimic Taxol-microtubules. The first is that the R5L mutation reduces the occupancy, or the amount of Tau bound at saturating conditions, without altering the affinity for Taxol-microtubules (32). The second is that the R5L mutation alters Tau-binding behavior and reduces Tau patches on Taxol-microtubules (32). These results indicate that dynamic microtubules in the presence of R5L-Tau depolymerize at a faster rate compared with WT-Tau. It is known that depolymerizing microtubules are comprised of GDP tubulin (60). Although the R5L mutation reduces Tau occupancy on dynamic microtubules, this is not likely the cause for the increase in microtubule shrinkage rate. When the same amounts of WT-Tau and R5L-Tau are bound to dynamic microtubules, R5L-Tau still results in an increase in microtubule shrinkage rate compared with WT-Tau.

Static Tau patches form on both dynamic microtubules at 37 °C and Taxol-microtubules at 25 °C (3, 31, 33), although the size and duration of these patches are decreased on dynamic microtubules relative to Taxol-microtubules. In addition, as with Taxol-microtubules (32), these patches are disrupted by introduction of the R5L mutation. There are fewer static binding events with the R5L mutant as well as a decrease in Tau fluorescence heterogeneity along the microtubule, indicating fewer Tau patches. Tau fluorescence heterogeneity was confirmed to correlate with Tau patches in control experiments with stabilized microtubules. Furthermore, Tau heterogeneity increased with Tau concentration, as Tau patches are known to form in a concentration-dependent manner on Taxol-microtubules (3, 31). Here, we show that 750 nM WT-Tau is bound more heterogeneously along dynamic microtubules compared with 300 nM WT-Tau. Moreover, the microtubule shrinkage rate can be seen to slow at Tau patches on dynamic microtubules in the presence of 750 nM WT-Tau, which have an overall slower shrinkage rate compared with dynamic microtubules in the presence of 300 nM WT-Tau. Altogether, the concentration dependence of WT-Tau, as well as the change in binding behavior observed with the R5L mutant, correlates the increase in microtubule shrinkage rate with a decrease in Tau patch formation on dynamic microtubules.

**Figure 8:**
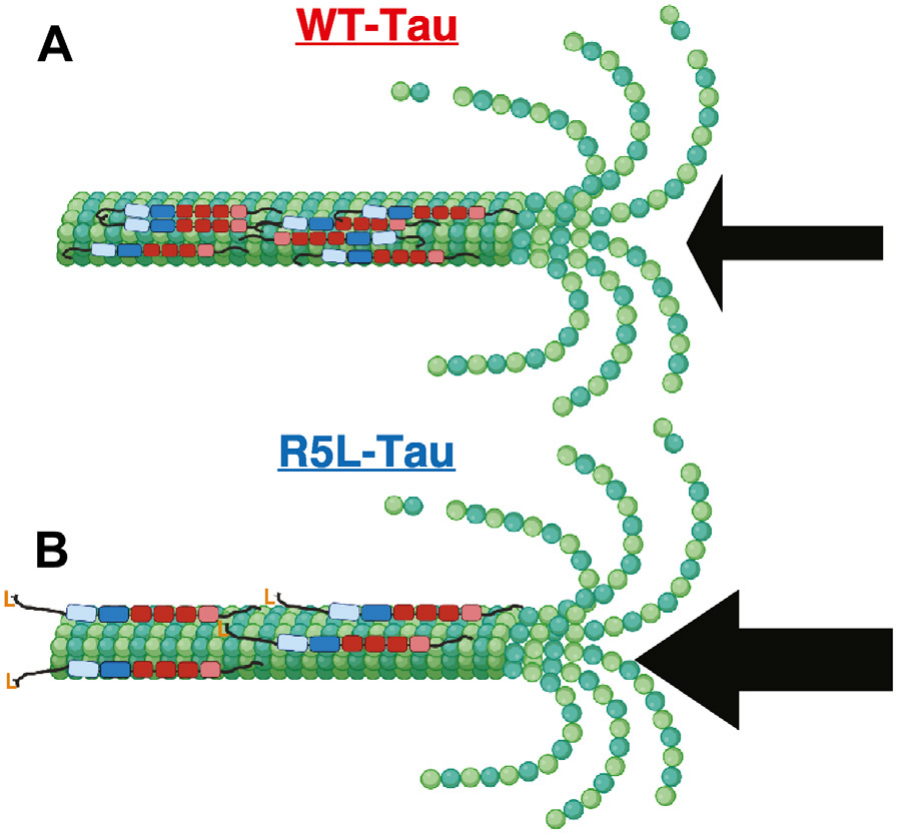
**Mechanistic model of the effect of the R5L mutation on microtubule dynamics.***A*, static WT-Tau patches decrease the inherent microtubule shrinkage rate (*thin arrow*). *B*, static Tau patches are disrupted upon introduction of the R5L mutation (shown as *orange* L), leading to an increase in microtubule shrinkage rate (*thick arrow*). Figure is created with biorender.com.

However, this still begs the question: how do Tau patches slow down the microtubule shrinkage rate? We hypothesize that Tau interprotofilament interactions, either with the microtubule or with other Tau molecules, increase lateral interactions across protofilaments, decreasing the microtubule shrinkage rate (Fig. 8) by one of several possible mechanisms. Tau patches could directly increase lateral protofilament interactions by inducing a structural change in tubulin, increasing the strength and/or number of bonds between tubulin dimers. In support of this hypothesis, recently, it has been shown that Tau patches are able to compact Taxol-microtubules (66). However, high-resolution structural studies of Tau bound with microtubules do not indicate structural changes in a native lattice (23). Alternatively, Tau patches could indirectly increase lateral protofilament interactions through molecular crowding effects, increasing the effective local concentration or restricting the rate of free tubulin diffusion away from the microtubule. Finally, Tau patches could indirectly increase lateral protofilament interactions by increasing Tau interactions with either tubulin subunits or other Tau molecules on adjacent protofilaments. Such a mechanism is consistent with recent NMR data that introduction of the R5L mutation leads to a loss of projection domain interactions in a microtubule-dependent manner (32). These interactions are proposed to be either with another Tau molecule, the microtubule, or more likely, a combination of transient interactions (32). Because of the highly flexible nature of the projection domain, it is feasible that a proportion of these interactions occurs across protofilaments and helps to stabilize the GDP lattice in dynamic microtubules, reducing the shrinkage rate.

Loss of interactions across protofilaments and increased microtubule shrinkage reduce overall microtubule assembly and stability, consistent with many models of tauopathies, but in a fundamentally different mechanism than previously studied mutations in the microtubule-binding region and proline-rich region (67). Those mutations, such as P301L and V337M, are proposed to lead to a loss of microtubule stability because of a reduction in Tau affinity for the microtubule (41, 68, 69). The R5L mutation, located in the projection domain, does not alter Tau affinity for the microtubule (32). Rather, in agreement with other studies that have shown the importance of the projection domain for Tau cohesive island formation on Taxol-microtubules (33), the R5L mutation disrupts microtubule-bound Tau patches (32). Here, we show that the R5L mutation increases the Tau-mediated microtubule shrinkage rate, indicating that disruption of Tau patches has a functional effect on Tau-mediated microtubule dynamics. Overall, despite the fact the R5L mutation is far removed from the microtubule-binding region, our results underscore the importance of the projection domain for Tau-mediated microtubule dynamics under both normal and pathological conditions.

## Experimental procedures

### Tau expression, purification, and labeling

Tau constructs for turbidity assay, FCS, and FRET were cloned into a lab-made pET-HT vector (a gift from L. Regan) with a tobacco etch virus–cleavable N-terminal His tag. Tau constructs were expressed and purified following previously published protocols (24). Cells were lysed by sonication, followed by centrifugation. The supernatant was incubated with nickel resin beads equilibrated in 50 mM Tris (pH 8), 500 mM NaCl, and 10 mm imidazole buffer for 1 h at 4 °C with gentle rotation. Tau was step-eluted with 400 mM imidazole. The imidazole concentration was reduced by several buffer exchange steps using Amicon concentrators (MilliporeSigma), and the protein was incubated with tobacco etch virus and DTT at 4 °C overnight or 37 °C for 4 h. The protein was run over a second nickel column to remove uncleaved Tau, the enzyme, and the tag, and then further purified by size-exclusion chromatography on a HiLoad 16/600 Superdex 200 Column (GE Life Sciences) in 25 mM Tris (pH 8), 100 mM NaCl, 1 mM EDTA, and 1 mM Tris(2-carboxyethyl)phosphine buffer.

To create tau constructs for site-specific labeling for FCS and smFRET experiments, QuickChange mutagenesis was used to remove the native cysteine of Tau (C322S; all numbering of amino acids is based on the longest isoform of Tau, as is the convention in the field) and to introduce new cysteines at the desired locations. For smFRET experiments, the labeling positions were chosen to span the domains of interest. For FCS measurements, the proteins were labeled at the native cysteine. To label, purified protein was reduced by incubation with 1 mM DTT for 30 min and exchanged into a labeling buffer (20 mM Tris [pH 7.4], 50 mM NaCl, and 6 M guanidine HCl) using Amicon concentrators (Millipore). To label a single cysteine for FCS measurements, Alexa 488 C_5_ maleimide (Invitrogen Molecular Probes) was added 5:1 dye:protein molar ratio and incubated with stirring overnight at 4 °C. For donor and acceptor labeling for smFRET measurements, the donor dye, Alexa 488 C_5_ maleimide, was added at 1:2 dye:protein molar ratio and incubated with stirring at room temperature for 1 h. The acceptor dye, Alexa 594 C_5_ maleimide, was added at a 5:1 dye:protein molar ratio and incubated with stirring overnight at 4 °C. Labeled protein was buffer exchanged into 20 mM Tris (pH 7.4) and 50 mM NaCl, and unreacted dye was removed by passing the labeled protein solution over a HiTrap Desalting Column (GE Life Sciences).

Tau constructs for TIRF microscopy were purified and characterized as described previously (1). Tau constructs were labeled using maleimide chemistry, conjugating Alexa-647 C_2_ maleimide or Alexa 488 C_5_ maleimide to the single cysteine residue as described previously (32).

### Turbidity assays

Polymerization assays were carried out on an infinite M100Pro Plate reader in 96 Flat Bottom Black Polystyrene plates (Thermo Fisher Scientific-Nunclon) by measuring light scattering intensity at 340 nm. About 10 μM tau was mixed with 20 μM tubulin on ice BRB80 buffer with 1 mM DTT, and 1 mM GTP was added to the sample and mixed five times immediately before transferring the sample to a warmed 96-well plate and starting the measurement at 37 °C. The intensities at 340 nM were recorded from the top of the sample recorded every 20 s. The scattering intensity of tubulin in the absence of tau was subtracted from the scattering intensity for tau-tubulin measurements. Three independent measurements were made for each tau construct, and the curves were averaged. The data were plotted and analyzed using GraphPad Prism 8 (GraphPad Software, Inc).

### FCS

FCS measurements were taken on a lab-built instrument based on an inverted Olympus Ix-71 Microscope (Olympus) (70). The laser power (488-nm diode-pumped solid-state laser, Spectra-Physics) entering the microscope was adjusted to 5 μW. Fluorescence emission was collected through the objective, filtered through a Z488RDC long-pass dichroic and an HQ600/200M bandpass filter, and focused onto 50 μm aperture optical fibers (OzOptics), directly coupled to avalanche photodiodes (PerkinElmer). A digital correlator (FLEX03LQ-12; Correlator.com) was used to generate the autocorrelation curves.

FCS measurements were carried out at ∼20 nM labeled protein in BRB80 buffer (80 mM Pipes, 1 mM EGTA, 1 mM MgCl_2_, pH 6.9) at 20 °C in chambered Nunc coverslips. To minimize protein adsorption, the coverslips were incubated with polylysine-conjugated PEG. Samples with tubulin were mixed gently five times and incubated for 5 min prior to measurement. For each measurement, 25 traces of 10 s each were collected and used to calculate the autocorrelation function, G(τ), as a function of the delay time τ. The autocorrelation curves were fit to an equation for diffusion in 3D of a single fluorescence species using lab-written scripts in MATLAB (The Mathworks):

(1) $$G\left(\tau \right)=\frac{1}{N}\times \frac{1}{1+\frac{\tau }{\tau_{D}}}\times \sqrt{\frac{1}{1+\frac{s^{2}\tau }{\tau_{D}}}}$$

where *N* is the average number of molecules in the focal volume, *s* is the ratio of radial to axial dimensions of the focal volume, and $\tau_{D}$ is the translational diffusion time. Each measurement was repeated three times to obtain an average $\tau_{D}$ values and SEM.

### FRET

All smFRET measurements were carried out on a MicroTime 200 inverse time-resolved confocal microscope (Picoquant). These measurements were made in the pulsed interleaved excitation FRET with excitation from 485 and 560 nm lasers pulsed at 40 MHz; power for both lasers was adjusted to enter the microscope at ∼30 μW. This mode of smFRET data collection allows for discrimination of low FRET efficiency (ET_eff_) events from those arising from molecules labeled only with a donor fluorophore (71). Fluorescence emission was collected through the objective and passed through a 485LP filter, and photons were separated by an HQ585LP dichroic in combination with ET525/50M and HQ600LP filters and collected by focusing on avalanche photodiodes.

smFRET measurements were carried out at ∼30 PM labeled protein in BRB80 buffer at 20 °C in polylysine-conjugated PEG-coated chambered Nunc coverslips, as noted previously. For each measurement, a single trace of 60 min was collected in 1 ms timebins. Events corresponding to transit of a protein were selected by applying a cutoff of 30 photons per event. Alternating laser excitation was used to identify events corresponding to protein labeled with both donor and acceptor probes and to exclude events arising from molecules labeled with only donor or acceptor fluorophores (72). For each photon burst containing both a donor signal and an acceptor signal, the ET_eff_ was calculated using SymphoTime64 software (Picoquant) with the following equation:

(2) $${\text{ET}}_{\text{eff}}=\frac{n_{A}}{n_{A}+\gamma n_{D}}$$

where *n*_*A*_ and *n*_*A*_ are the number of photons per burst (corrected for detector crosstalk) in the acceptor and donor channels, respectively, and *γ* is a correction factor for the difference in detection efficiency of the donor channel relative to the acceptor channel.

The individual ET_eff_ values were compiled as histograms, which were fit using either a sum of two Gaussian distributions or a single Gaussian with a *y*-axis offset on Prism 8. These simply account for the differing backgrounds in the sample, and the method of fitting does not impact the mean value of the peak arising from the protein. Each sample was measured three times independently.

### Tubulin purification and preparation of GMPCPP seeds

Tubulin was purified from bovine brain obtained from Vermont Livestock Slaughter & Processing (Ferrisburgh) as described previously using High Molarity Pipes buffer (1 M Pipes, 20 mM EGTA, 10 mM MgCl_2_, pH 6.9) (73). Microtubule seeds stabilized with GMPCPP sodium salt (Jena Bioscience) were prepared by incubating 10 μM tubulin with 5% Alexa-488 or Alexa-647 tubulin (Cytoskeleton) and 1 mM GMPCPP. After 20 min at 37 °C, an equal volume of 5% labeled tubulin was added and allowed to incubate for 15 min. Samples were centrifuged at 80,000 rpm in an Optima TLX Ultracentrifuge (Beckman) with TLA100 rotor equilibrated at 37 °C for 7 min. The pellet was resuspended in warm BRB80 flash frozen and stored at −80 °C until use.

### TIRF microscopy

TIRF microscopy experiments were performed at 37 °C on an inverted Eclipse Ti-E microscope (Nikon) with 100× Apo TIRF objective lens (1.49 numerical aperture) and dual iXon Ultra Electron Multiplying CCD cameras, running NIS Elements version 4.51.0. Experiments were performed in TIRF assay buffer (TAB) containing (BRB80, 10 mM DTT, and oxygen scavenger system [0.067 mg/ml glucose oxidase, 0.045 mg/ml catalase, and 5.8 mg/ml glucose; Sigma–Aldrich]). Glass coverslips, prepared as described (74), were incubated with 33 μg/ml monoclonal anti-beta III tubulin antibodies (Sigma–Aldrich) and blocked with 1% pluronic F-127 (Sigma–Aldrich), and washed with three chamber volumes of TAB. GMPCPP-seeds were added to the chamber at 15 nM and allowed to incubate for 15 min followed by a chamber wash with TAB to remove nonadherent seeds. The final solution containing 300 to 750 nM Tau (WT-Tau or R5L-Tau), 5 μM tubulin, 1 mM GTP, in TAB supplemented with 0.1% methyl cellulose (Sigma–Aldrich) and 0.5 mg/ml bovine serum albumin (Thermo Fisher Scientific) was added and allowed to incubate for 10 min at 37 °C before imaging. The proportion of labeled:unlabeled protein (Tau or tubulin) and imaging conditions was dependent on experiment.

For microtubule dynamics experiments, GMPCPP seeds were labeled with 5% Alexa-647, free tubulin–contained 5% Alexa-488 tubulin, and Tau (WT-Tau or R5L-Tau) was unlabeled. After the 10 min incubation, Alexa-488-labeled dynamic microtubules were excited with a 488 laser (5%), passed through 525/50 filter while GMPCPP-seeds were excited with a 640 laser (5%), passed through a 655 long-pass filter. Both were imaged at a 2 s interval with a 300 ms exposure for 10 min. Imaging did not exceed 40 min. Images were quantified from kymographs generated in FIJI (ImageJ; National Institutes of Health). From kymographs, the growth rate, shrinkage rate, and catastrophe frequency were measured.

For Tau single-molecule mobility experiments, GMPCPP seeds were labeled with 5% Alexa-488, free tubulin–contained 10% Alexa-488 tubulin, and 750 nM unlabeled Tau (WT-Tau or R5L-Tau) was spiked with 300 pM Alexa-647-labeled Tau to visualize individual molecules. Alexa-488-labeled seeds and/or microtubules were excited with a 488 laser (5%), passed though 525/50 filter, whereas Tau (WT-Tau or R5L-Tau) was excited with a 640 laser (15%), passed through a 655 long-pass filter. Both Tau and microtubules were imaged with 100 ms exposure. Tau was imaged at 10 frames/s, whereas microtubules were imaged at 2 frames/s for 30 s. Single-molecule data were analyzed as described previously (32) using MATLAB to determine static:diffusive equilibrium, diffusion coefficient, and dwell times.

For Tau occupancy experiments, GMPCPP seeds were labeled with 5% Alexa-488, unlabeled free tubulin, and 750 nM Alexa-647 Tau (WT-Tau or R5L-Tau). After the 10 min incubation, GMPCPP-seeds were excited with a 488 laser (5%), passed though 525/50 filter, whereas Tau (WT-Tau or R5L-Tau) was excited with a 640 laser (1%), passed through a 655 long-pass filter. Both Tau and microtubules were imaged with 100 ms exposure. Tau was imaged at 10 frames/s, whereas microtubules were imaged at 2 frames/s for 30 s. Occupancy was determined by measuring the normalized average fluorescence intensity of Tau (WT-Tau or R5L-Tau) on microtubules, excluding seeds.

For Tau patch experiments, GMPCPP seeds were labeled with 5% Alexa-488, unlabeled tubulin, either 250 nM or 650 nM unlabeled Tau (WT-Tau or R5L-Tau), and 100 nM Alexa-647-labeled Tau (WT-Tau or R5L-Tau). After the 10 min incubation, Alexa-488-labeled seeds and microtubules were excited with a 488 laser (5%), passed though 525/50 filter, whereas Tau (WT-Tau or R5L-Tau) was excited with a 640 laser (10%), passed through a 655 long-pass filter. Both Tau and microtubules were imaged with 100 ms exposure at 10 frames/s for 1 min. Tau patch analysis was performed using custom MATLAB script by measuring relative differences in average Tau intensity along the microtubule.

## Data availability

All data are found in the article or supporting information.

## Supporting information

This article contains supporting information.

## Conflict of interest

The authors declare that they have no conflicts of interest with the contents of this article.
