# Supplementary material for: The N-terminal disease–associated R5L Tau mutation increases microtubule shrinkage rate due to disruption of microtubule-bound Tau patches
Source: J Biol Chem. 2022 Sep 24;298(11):102526. doi: 10.1016/j.jbc.2022.102526 (PMC9589210; doi:10.1016/j.jbc.2022.102526)
Supplement: Supporting Information [file mmc5.docx]

**Supporting Information**

**Title**

The N-terminal disease-associated R5L Tau mutation increases microtubule shrinkage rate due to disruption of microtubule-bound Tau patches

**Authors**

Alisa Cario^1^, Sanjula P. Wickramasinghe^2^, Elizabeth Rhoades^2,3^, Christopher L. Berger^1,*^

1-Department of Molecular Physiology and Biophysics, University of Vermont, Burlington, VT 05405

2- Biochemistry and Molecular Biophysics Graduate Group, Perelman School of Medicine, University of Pennsylvania, Philadelphia, Pennsylvania

3- Department of Chemistry, University of Pennsylvania, Philadelphia, Pennsylvania

*Christopher L. Berger Dept. of Molecular Physiology and Biophysics, University of Vermont, 149 Beaumont Ave., Burlington, VT 05405 Tel: 802-656-0832; Fax: 802-656-0747;

**Email:** [cberger@uvm.edu](mailto:cberger@uvm.edu)

**Materials Included:**

Figures S1-S6

Legends for Movies S1-S4

**Fig. S1**. **Diffusion times of WT-Tau and R5L-Tau.** Raw diffusion times of 20 nM WT-Tau (red) or R5L-Tau (blue) at varying concentrations of tubulin determined via FCS. Data are fit to a standard binding model as described in Methods. The apparent offset between WT-Tau and R5L-Tau is due to difference in diffusion times in the absence of tubulin. Once normalized to the absence of tubulin, there is no difference between WT-Tau and R5L-Tau (as seen in Fig 2A). Data are mean ± SD (N = 3).

**Fig. S2. Time microtubules spend growing and shrinking A**. Time of microtubule growth at plus ends comparing WT-Tau (red, 228.3 ± 158.7 sec, N = 119) and R5L-Tau (blue, 281.5 ± 186.3 sec, N = 96 ). Data are mean ± SD. Statistical analysis was performed using student’s t-test (* p < 0.05). **B**. Time of microtubule shrinkage at plus ends comparing WT-Tau (red, 15.38 ± 12.28 sec, N = 69) and R5L-Tau (blue, 11.78 ± 7.46 sec, N = 46). Data are mean ± SD. Statistical analysis was performed using student’s t-test (* p < 0.05).

**Fig. S3. Change in fluorescence intensity of WT-Tau along stabilized microtubules.** WT-Tau fluorescence heterogeneity along stabilized microtubules comparing Tau along Taxol-microtubules (plum, 0.31 ± 0.06, N = 77) and GMPCPP-microtubules (purple, 0.22 ± 0.08, N = 78). Data are median ± 95% CI. Statistical analysis was performed using Mann-Whitney test (* p < 0.001).

**Fig. S4. Kymographs of fluctuations in microtubule depolymerization rate in the presence of Tau patches.** Left to right: Representative kymographs indicating increasing heterogeneity in depolymerization rate corresponding with increasing Tau patches. All experiments were done in the presence of 750 nM WT-Tau (100 nM Alexa-647 WT-Tau + 650 nM WT-Tau). Patches are shown in magenta and microtubules are shown in green. White arrows denote periods of decreased shrinkage rates.

** Fig. S5. Kymographs of WT-Tau and R5L-Tau binding on dynamic microtubules.** Kymographs of WT-Tau (left) and R5L-Tau (right) showing both static (top) and diffusive (bottom) on dynamic microtubules with 300 pM labeled Tau and 750 nM total Tau.

**Fig. S6. Histograms of single molecule mobility data A.** Histograms of diffusion coefficients of WT-Tau (red) and R5L-Tau (blue). **B.** Histograms of static dwell times of WT-Tau (red) and R5L-Tau (blue). **C.** Histograms of diffusive dwell times of WT-Tau (red) and R5L-Tau (blue).

**Movie S1.** WT-Tau-mediated microtubule dynamics. Representative movie of dynamic microtubules (green) grown from GMPCPP seeds (magenta) in the presence of 750 nM WT-Tau (unlabeled). Microtubules imaged at 0.5 frames/sec. Scale bar = 2 μm.

**Movie S2.** R5L-Tau-mediated microtubule dynamics. Representative movie of dynamic microtubules (green) grown from GMPCPP seeds (magenta) in the presence of 750 nM R5L-Tau (unlabeled). Microtubules imaged at 0.5 frames/sec. Scale bar = 2 μm.

**Movie S3.** WT-Tau binding behavior on dynamic microtubules. Representative movie of 750 nM WT-Tau binding behavior with 300 pM Alexa 647 labeled WT-Tau to image individual molecules. Tau imaged at 10 frames/sec. Scale bar = 2 μm.

**Movie S4.** R5L-Tau binding behavior on dynamic microtubules. Representative movie of 750 nM R5L-Tau binding behavior with 300 pM Alexa 647 labeled R5L-Tau to image individual molecules. Tau imaged at 10 frames/sec. Scale bar = 2 μm.
